# Embedding research within occupational therapy pre-registration training: A concept mapping study engaging staff and student voices

**DOI:** 10.1177/03080226241253102

**Published:** 2024-05-22

**Authors:** Katie L Hackett, Helen Atkin, Sureshkumar Kamalakannan, Savannah Murray Mendes, Julie Anne Lowe, Phillip Whitehead, Gemma Bradley

**Affiliations:** 1 Department of Social Work, Education and Community Wellbeing, Faculty of Health and Life Sciences, Northumbria University, Newcastle upon Tyne, UK; 2 King’s College Hospital, London, UK; 3 Population Health Sciences Institute, Newcastle University, Newcastle upon Tyne, UK

**Keywords:** Occupational therapy, education, evidence-based care, students, mixed methods, research capacity building

## Abstract

**Introduction:**

Occupational therapists require research and evaluation skills to deliver evidence-based care, making research education integral to their training. We aimed to develop a student and staff-informed strategy to further embed research into the occupational therapy programmes and enhance the research culture at a United Kingdom Higher Education Institution.

**Method:**

We used group concept mapping to gather ideas from students and staff on how to embed research and improve research culture within the occupational therapy programmes at a United Kingdom Higher Education Institution. Participants generated, sorted and rated ideas for importance and success. We analysed the data to create a concept map and identified rating values for the themed clusters and their ideas.

**Results:**

The concept map contained four themed clusters of ideas: Wider research community, Integration of research into the core programme, Advanced research support and capacity building and Research awareness. Successes and improvement targets were identified within each cluster.

**Conclusion:**

Group concept mapping provided a structured and comprehensive method to develop a strategy for embedding research and fostering a research culture in occupational therapy programmes at a higher education institution. The four-themed concept map and identified priority targets serve as a foundation for implementing the strategy and improving research integration in occupational therapy education.

## Introduction and literature review

Occupational therapists in the United Kingdom (UK) must comply with the Health and Care Professions Council’s (HCPC) standards to obtain and maintain their registration and practice proficiently (HCPC, 2023). These standards specify that at the point of registration, occupational therapists must ‘engage in evidence-based practice’ (#11.1), ‘recognise the value of research to the critical evaluation of practice’ (#13.9), ‘critically evaluate research and other evidence to inform their own practice’(#13.10) and ‘engage service users in research as appropriate’ (#13.11). Given the centrality of research and evidence-based practice in the profession (RCOT, 2019c), pre-registration training must equip students with the necessary skills to meet these standards (RCOT, 2019b).

In England, policy drivers (NHS England, 2017) such as *Allied Health Professions Deliver* (NHS England, 2022), the *Allied Health Professions’ Research and Innovation Strategy for England* (Health Education England, 2022) and the *Multi-professional Practice-based research capabilities framework* (NHS England, 2024) have emphasised the need for allied health professionals (AHPs) to be evidence-based practitioners, with their practice guided by research, innovation and quality improvement (QI), which should be embedded into job descriptions across all stages of their career (Health Education England, 2022). The *NHS Long Term Plan* also emphasises the use of systematic QI methods to achieve its goal of promoting innovation and facilitating the implementation of effective changes (NHS, 2019). While there are differences in approaches to achieving evidence-based practice, research and QI, there are cross-cutting skills, such as formulating appropriate questions, critically appraising literature and collecting data to evaluate patient outcomes (Kawar et al., 2023). The Royal College of Occupational Therapists (RCOT) added to these drivers and collaborated with the occupational therapy workforce to set research priorities and identify their own research priorities (RCOT, 2019c). To ensure that occupational therapists can contribute to these ambitions and meet the required professional standards, they need foundational knowledge of research and evaluation skills. A previous review of research development activities in the UK found that workplace culture did not promote research-related activities (White et al., 2013). Subsequently, Di Bona et al. (2019) proposed a four-step method for registered occupational therapists to develop research skills and pursue a clinical academic career, and other frameworks for embedding a culture of research in clinical practice among AHP groups have been developed (Slade et al., 2018). However, to develop a research culture within the profession, research awareness and skills should be developed early in the career journey. Recent research in a Norwegian occupational therapy programme suggests introducing research earlier at the pre-registration level through research-informed teaching, and developing students’ research skills could be a promising approach to promote evidence-based practice and research skills from the outset (Helgoy et al., 2020).

The *RCOT Career Development Framework* allows pre-registration and qualified occupational therapists to evaluate and map their learning needs within each of four interdependent pillars of practice for successful career development: including the Evidence, Research and Development Pillar. At Northumbria University, students are encouraged to reflect on their growth in these areas during practice placements and throughout their studies including an assessment prior to course completion. Although research is a part of the occupational therapy teaching programme at Northumbria University, a specific strategy to support staff and students in publishing their research, obtaining grants and developing future clinician researchers is lacking. *The Connected Curriculum Framework* (Fung, 2017) is an example of how research-based education can be developed in higher education. It incorporates a model placing learning through research and enquiry at its centre and includes six dimensions emphasising the need for connectivity in specific areas, such as students connecting with the research of their teaching staff and the wider institution (Fung, 2017).

The Health Education England Allied Health Professions’ Research and Innovation Strategy (Health Education England, 2022) identifies the interconnection between capacity, capability, context and culture to deliver transformational growth in research and innovation for AHPs, including occupational therapists. Research culture, where AHPs hold the perception and expectation that ‘research and innovation is everyone’s business’ (Health Education England, 2022), is recognised as influencing the challenge of embedding research in practice-based roles (Comer et al., 2022). In addition, NHS England (2024) emphasises frameworks to support the translation of research capabilities across all levels of practice.

A recent overview of systematic reviews focussing on interventions, methods and outcome measures used in teaching evidence-based practice to healthcare students (Nielsen et al., 2024) highlights wide variation in these practices and reflects difficulty in making any best practice recommendations due to low quality evidence. Some of the most common methods for teaching research to undergraduate health students include research courses and workshops and collaborations with clinical practice, with methods such as journal clubs and embedded librarians less commonly discussed (Larsen et al., 2019). However, it presents a challenge to ascertain whether specific teaching approaches are more effective than others, in part due to wide heterogeneity of outcomes measured and wide variation in educational systems and programmes (Nielsen et al., 2024).

At Northumbria University, first-year pre-registration occupational therapy students are introduced to academic writing and literature searching techniques, and second-year students learn critical appraisal and evidence-based practice (first year for MSc students). In their final year, students conduct a systematic literature review for their dissertation. MSc students can also choose to conduct empirical studies. However, it is unclear as to whether the content and timing of such research-related teaching is enough to firmly embed research within the programmes and to foster a research culture among students and staff. To support the RCOT ambitions (RCOT, 2019a) and respond to policy drivers (Health Education England, 2022; NHS England, 2022, 2024), it is essential that we evaluate our programmes and identify improvement targets to normalise research and develop confidence and skills among occupational therapists entering the workforce.

Co-creation with occupational therapy programme staff and students is key to developing a feasible and relevant strategy addressing high-priority areas. Engaging students and staff in in the process promotes learning and research experiences (Dollinger and Lodge, 2020) and ensures that the strategy is tailored to the programme’s needs and resources.

Group concept mapping (GCM) is a mixed-methods approach that combines group processes and multivariate statistical analyses to create visual representations of stakeholders’ opinions (Trochim, 1989). Participants provide brainstormed ideas, sort these ideas and assign priority values. The resulting concept map and go-zone plot can be used for planning or evaluation (Kane and Trochim, 2007). The advantage of this approach is that it can engage a large and diverse community of interest, integrate their knowledge and develop a co-authored strategy without requiring consensus (Kane and Rosas, 2018). A comprehensive examination of pooled study data has revealed robust internal representational validity alongside very high sorting and rating reliability estimates (Rosas and Kane, 2012). GCM has been used in various medical and nursing education settings (Hagell et al., 2016; Kilty et al., 2017), to identify research training needs in practitioners and researchers (Tabak et al., 2017) and to develop and evaluate occupational therapy interventions (Fraser et al., 2022). The rating applied to the statements generated during the process can be used to evaluate the current state and identify future improvement targets (Robinson et al., 2023).

We aimed to involve students and staff at Northumbria University in creating a concept map to inform a strategy to further embed research within occupational therapy programmes and to enhance the research culture. Our objectives were to engage staff and students to identify and prioritise needs and ideas, assess their current success and strategically plan future targeted improvements by focussing on high priority needs which were not being successfully met.

## Method

### Design

We used GCM methodology (Kane and Trochim, 2007) to explore how research could be better embedded within the occupational therapy programmes and how the research culture at a higher education institution (HEI) can be enhanced by generating a student and staff co-authored concept map and a go-zone – a bivariate scatter plot – to depict important areas of success and targets for improvement.

### Setting and timing of the study

Data collection took place at a post-1992 university in England, which offers pre-registration training for occupational therapists through a 3-year BSc (Hons) or a 2-year MSc degree. Data collection was conducted prior to the COVID-19 pandemic and to the launch of a new degree apprenticeship programme.

### Participant groups

Participants were recruited from two stakeholder groups: (1) students from the BSc and MSc occupational therapy programmes and (2) staff aligned with the programmes. To ensure that we incorporated the viewpoints and value ratings of all students and staff, we invited all current staff and students to participate via email and verbal announcements during student lectures and at a staff meeting. GCM studies can range from 20 to over 600 participants (Rosas and Kane, 2012).

### The GCM stages

Informed consent was provided by the participants prior to completing any of the concept mapping tasks. Participants were given the option of completing brainstorming, sorting and rating activities online (via the CS Global MAX™ platform, which is designed for GCM projects), or with paper copies if preferred. Because the sorting task can be time-consuming, participants were given the option of completing the rating activity without doing the sorting task. The sorting task was available online as it was considered more convenient for participants to complete digitally than with printed cards. Two follow-up reminder emails were sent to potential participants. Data collection took place over a semester.

The GCM study comprised five distinct stages: brainstorming (idea generation), statement reduction, sorting, rating, analysis and interpretation, which are detailed below.

#### Stage 1: Brainstorming

Participants were provided with an incomplete sentence/focus prompt, which they were asked to complete as many times as they could think of an idea. The wording of the focus prompt was intended to identify specific ways in which research could better be embedded within the occupational therapy programmes. The focus prompt provided to the participants was:

One specific action which could be taken to embed research within the Occupational Therapy programmes at Northumbria University is. . . . . . . . . . . .

Participants were also provided with the following text to help them generate ideas:

If you are stuck with ideas, you may consider the following:

• *Specific support you would like with research*

• *A specific action which might contribute towards a culture of research*

This process generated a list of statements from participants during this stage of the study. Although participants completed the brainstorming independently and anonymously, any participant who completed this task online could see the ideas that had been proposed by participants who had already completed the online brainstorming activity. The ideas provided on paper were uploaded to the CS Global MAX™ platform by (KLH), and the full list of ideas was exported into an Excel document in preparation for Stage 2.

#### Stage 2: Statement reduction

To ensure that the ideas generated in Stage 1 were unique and representative, duplicate statements were removed and overlapping ideas were combined at an involvement meeting with programme staff and author discussions. The final refined list of 72 statements was reviewed by the project team for syntax and readability. This made the statement list manageable for Stages 3 and 4.

#### Stage 3: Sorting task

Each statement was assigned a random number between 1 and 72 using a random number generator in the CS Global MAX™ platform, and participants were asked to sort the statements by grouping similar ones into piles and giving each pile a name that reflected its themed content.

#### Stage 4: Rating activity

Participants rated the importance and current success of each statement on a five-point Likert scale (1 = relatively unimportant/need not being met at all; 5 = extremely important/need is successfully met).

#### Stage 5: Data analysis

Data were analysed using the CS Global MAX™ platform. Sorted statements were converted into a binary similarity matrix for each participant and then combined into a summed square matrix (Kane and Trochim, 2007). Multidimensional scaling (MDS) was used to create a two-dimensional point map representation of the data, which depicts the numbered statements and the relationships between them. Simply put, statements that are sorted together frequently by participants (as they are considered to be conceptually similar) will be located near each other on the point map, and statements that are infrequently sorted together will be located further apart. The relationship between the point map and the summed square matrix was assessed using the stress value, which is generated during MDS (Kruskal and Wish, 1978). Stress measures the extent to which distances on the two-dimensional map are discrepant from the values inputted into the similarity matrix with a lower value indicating a better fit (Kane and Trochim, 2007). The acceptable stress value range for GCM projects is 0.205–0.365 (Rosas and Kane, 2012). Therefore, concept maps with a stress value within this range or below will demonstrate an acceptable goodness of fit between the inputted matrix data (how the statements were sorted by the participants) and the resulting point map.

Next, we applied hierarchical cluster analysis using Ward’s method (Ward, 1963) to divide the point map into non-overlapping clusters of aggregated ideas reflecting different conceptual themes (Kane and Trochim, 2007). Members of the project team combined clusters one at a time, starting with 20 clusters, and discussed the statements within each cluster at each iteration to ensure that they conveyed the overall theme. We continued combining clusters until it no longer made sense to proceed to the next iteration and decided on a final cluster solution through discussion. The platform suggested labels for each themed cluster based on the names provided by the participants during the sorting exercise. The authors agreed the final cluster names, based on these suggestions.

The mean importance and success ratings were calculated at the cluster level and compared at the individual statement level within go-zones. Go-zones are plots that show the rating scores, divided into quadrants based on mean values of importance and current success dimensions. Statements with high ratings for both importance and success fall within the top-right quadrant. These are important ideas that are being successfully met. Statements in the lower right quadrant are important ideas that are not currently being successfully met, or in need of improvement (Robinson et al., 2020). These were used to inform and prioritise improvements that could be made within the occupational therapy programmes to embed research and enhance the research culture and were considered as targets for improvement. Although statements in the lower left quadrant (less important ideas which are not being successfully met) would be considered a lower priority, they could also be used to inform future overall improvements after the improvement targets are addressed.

#### Stage 6: Interpreting the maps

The research group and programme staff discussed the resulting concept maps. They used the mean cluster values and the go-zone to identify priority areas to inform an improvement strategy.

### Ethical approval

The study was reviewed by Northumbria University Ethics Committee (ref 10715) before recruitment and data collection began. If potential participants were interested in the study, they were provided with a participant information sheet and given the opportunity to ask the research team any questions about it prior to providing their written or digital consent and completing any of the activities.

### Service user involvement

A pre-registration occupational therapy student and occupational therapy programme staff collaborated to design and conduct this study. This project team also analysed and interpreted the data. In addition, occupational therapy programme staff (both participants and non-participants) were involved in the decision-making during the statement reduction process.

## Results

A total of 102 participants (94 students and eight programme staff members) participated in the brainstorming activity. As this stage was anonymous, it was not possible to determine whether the same participants took part in the subsequent tasks. Of the 86 participants (78 students and eight staff) who took part in the sorting and rating tasks, five students and eight programme staff completed the sorting task, 67 students and eight staff completed the importance rating task, and 61 students and seven staff completed the success rating. A breakdown of the completion rates of the separate activities is presented in Table 1.

**Table 1.** The breakdown of participants taking part in each stage of the concept mapping exercise.

Participant group	Brainstorming	Sorting	Rating: Importance	Rating: Success
Student BSc Year 1	32	1	19	13
Student BSc Year 2	24	0	10	8
Student BSc Year 3	23	2	23	26
MSc Year 1	2	2	8	7
MSc Year 2	13	0	7	7
Programme Staff member	8	8	8	7
Total	102	13	75	68

**Figure 1.**
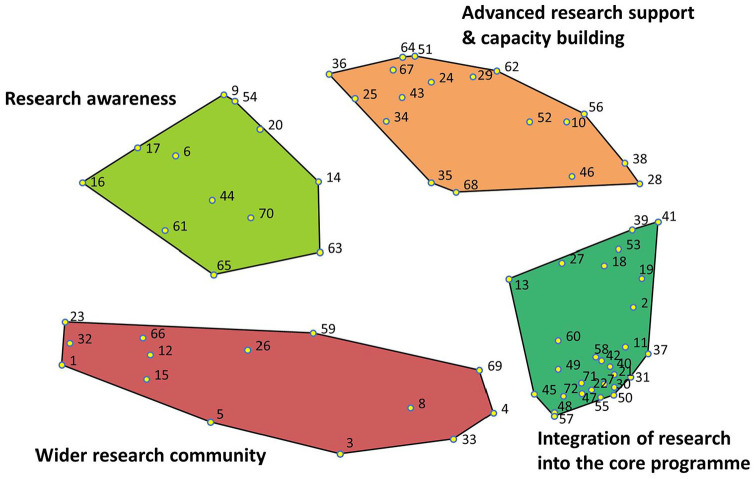
Concept map depicting the numbered statements within their themed clusters.

**Figure 2.**
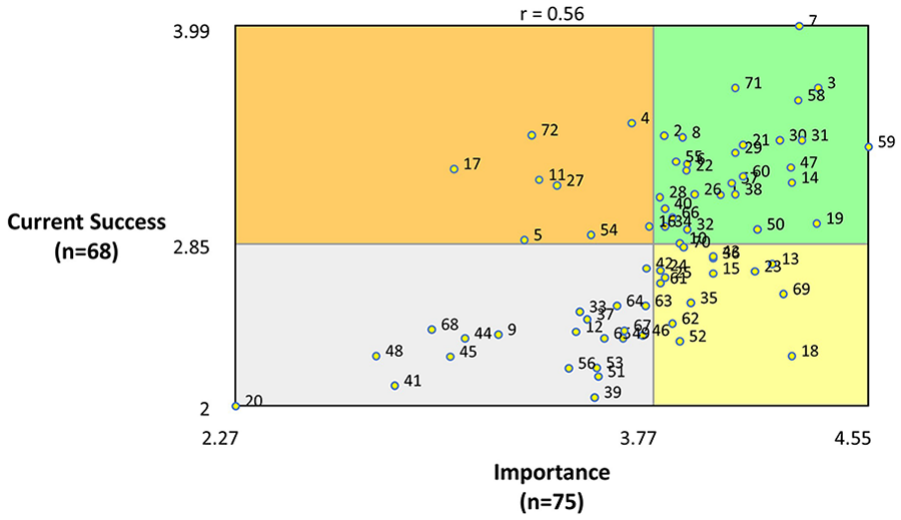
Bi-variate scatter plot showing go-zone analysis of item ratings for importance (*x*-axis) and current success (*y*-axis). The top-right quadrant depicts statements that have been rated above average for importance and success. The bottom right quadrant depicts numbered statements that are above average for importance and below average for success and are therefore targets for improvement.

**Figure 3.**
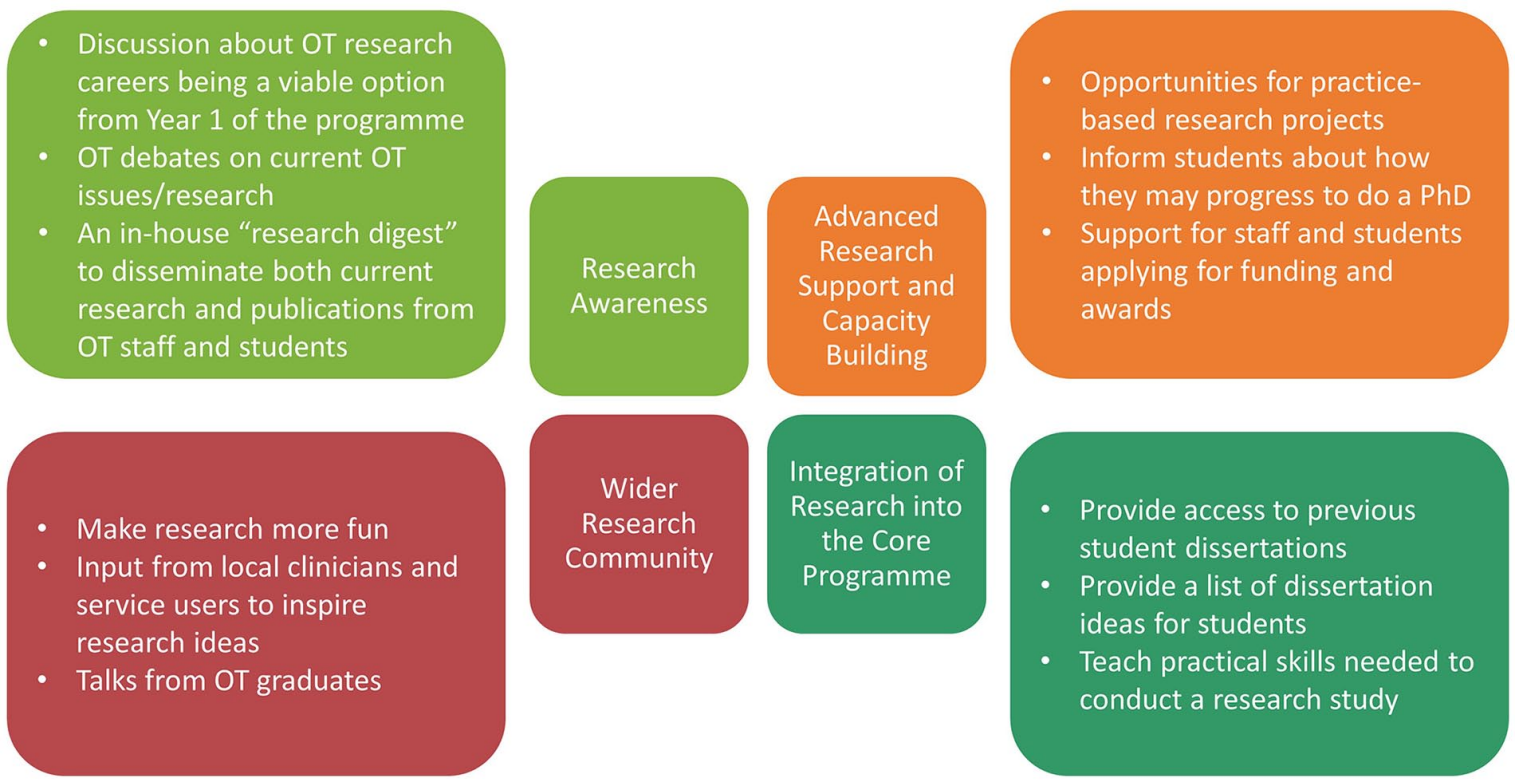
A simplified model of the concept map depicting the four conceptual themes and specific targets for improvement within each.

The brainstorming activity generated 241 statements. Some statements were split, as they contained more than one concept, which resulted in 245 statements. These were subsequently reduced to 72 unique statements after eliminating duplicate and six redundant ideas that were not directly related to the focus prompt, including ‘look at groups while talking to the student’. MDS analysis resulted in a stress value of 0.2759 after 12 iterations, indicating a good fit (Rosas and Kane, 2012). Following hierarchical cluster analysis, a final four-cluster solution was determined. The cluster domains were: (1) Wider Research Community, (2) Integration of Research into the Core Programme, (3) Advanced Research Support and Capacity Building and (4) Research Awareness. The mean importance and current success rating scores for each cluster are shown in Table 2, along with their included statements and corresponding rating scores. The concept map depicting the numbered statements within their named themed clusters is shown in Figure 1. A bivariate scatter plot showing the go-zone analysis of the importance and current success ratings for all statements is shown in Figure 2, and a simplified model of the four conceptual themes is shown in Figure 3.

**Table 2.** Mean importance and success ratings for the clusters and their corresponding statements.

Themed Clusters and their Corresponding Numbered Statements		Importance (1–5)	Current Success (1–5)
**1. Wider research community cluster**		**3.91**	**2.98**
59^†^	Give examples of where research has changed occupational therapy practice	4.55	3.35
3^†^	Have patient/service user involvement in the teaching of research	4.36	3.66
69^‡^	Make research more fun	4.24	2.58
23^‡^	Input and inspiration from local clinicians and service users to help develop research ideas	4.14	2.70
1^†^	Encourage occupational therapy staff and students to make links with other disciplines	4.01	3.10
15^‡^	To have talks from external OT graduates	3.99	2.69
26^†^	To prioritise and focus on the Royal College of Occupational Therapists research objectives	3.92	3.11
32	Invite practitioners from a range of clinical backgrounds to talk about their research	3.89	2.92
8	Lectures with researchers to explain their studies and processes	3.88	3.40
66	Guest speakers to talk about their research	3.84	2.98
4	Lecturers to use their own research data when teaching statistics and qualitative data analysis	3.69	3.48
33	To collate research evidence on relevant topic whilst on placement	3.51	2.49
12	To have talks from internal and external PhD candidates	3.49	2.39
5	Additional lectures from researchers from different disciplines	3.31	2.87
**2. Integration of research into the core programme cluster**		**3.82**	**2.97**
19^‡^	Have extra timetabled research sessions for those who may want additional support	4.36	2.95
31^†^	Teaching on academic writing	4.31	3.39
7^†^	Staff to provide direction to useful research resources, books and chapters	4.30	3.99
58^†^	Access to online research tutorials/lectures	4.29	3.60
18^‡^	Provide access to examples of previous dissertations	4.27	2.26
47^†^	Early use of critical appraisal tools within the programme	4.27	3.25
30^†^	Use visual and interactive activities to teach research methods to aid understanding	4.23	3.39
13^‡^	To provide students a list of research project options to research for a dissertation	4.20	2.74
50^‡^	Teach practical skills to help students learn how to design and carry out a research project, for example, preparing interview schedules	4.15	2.92
21^†^	Teach information retrieval strategies	4.09	3.36
60^†^	Demystify research and make it more accessible	4.09	3.20
71†	PowerPoints for student lectures to include detailed information and references	4.07	3.66
57^†^	Incorporate research examples throughout all modules of the programme	4.05	3.16
22^†^	Incorporate practical, primary data collection tasks within research methods teaching	3.89	3.23
55^†^	Practical tasks to learn and understand the research process	3.85	3.28
40	Teaching on the ethical review process	3.81	3.03
2	Small group research tutorials	3.81	3.41
42	To provide students with specific research terms list/glossary	3.75	2.72
49	Do a mock research study so students are more aware of what research involves	3.66	2.35
53	Opportunity for BSc OT students to complete an empirical research project as their Year 3 dissertation	3.57	2.20
39	An optional advanced module for people interested in research	3.56	2.05
37	In depth study of how to carry out peer review	3.53	2.45
27	Small group research projects/assignments	3.42	3.15
11	Research ‘homework’ to promote independent reading and reflection	3.36	3.18
72	Have a separate research module	3.33	3.42
45	Complete mini research projects throughout the course	3.04	2.26
41	A regular journal club	2.84	2.11
48	Get rid of separate research modules	2.77	2.26
**3. Advanced research support and capacity building cluster**		**3.77**	**2.64**
29^†^	Sessions where staff and students could present their research ideas and gain feedback from both staff and students	4.07	3.32
38^†^	Support to develop research ideas	4.07	3.11
36^†^	Offer research drop-ins for staff and students to discuss their research ideas	3.99	2.77
43^†^	Protected time for research activities	3.99	2.78
35^‡^	Opportunities for practice-based research projects	3.91	2.54
10^†^	Research mentors for staff and students	3.87	2.85
52^‡^	Inform students how they may progress to completing a PhD	3.87	2.34
62^‡^	Additional support for staff and students who wish to apply for funding and awards	3.84	2.43
25^†^	Clear signposting to research opportunities both within Northumbria University and outside of the organisation	3.81	2.67
34^†^	Hold an annual event for all students, staff and clinicians presenting current and completed projects and generating ideas for new projects	3.81	2.94
24^†^	Provide opportunities for students to be involved in planning and designing research projects	3.80	2.71
28^†^	To assign a dedicated research supervisor to every student	3.79	3.09
46	Put on optional workshops to help support students wishing to submit abstracts to conferences or publish their work	3.73	2.37
67	Opportunities for students to be able to work in partnership with staff and PhD students on projects	3.67	2.39
64	Postgraduate research study space/lab	3.64	2.52
51	Opportunities for students to undertake research internships	3.57	2.15
56	Introduce a research ‘buddy’ programme with other students/researchers at Northumbria University	3.47	2.20
68	Free up time to do research at the expense of another topic	2.97	2.40
**4. Research awareness cluster**		**3.51**	**2.72**
14^†^	Ensure published Northumbria University research is readily accessible	4.27	3.17
6^†^	Generate discussions on potential areas for future research	3.89	3.26
70^†^	Organise Q&A sessions from senior researchers for staff and students	3.88	2.83
61^‡^	Discuss research careers for occupational therapists as being a viable option from year 1 of the programme	3.80	2.64
16^†^	Research students’ experience on the programme at Northumbria University	3.76	2.94
63^‡^	OT debates to create discourse around current OT issues/research	3.74	2.52
65^‡^	An in house ‘research digest’ that compiles relevant and recent research undertaken by staff/students from the occupational therapy programme	3.59	2.35
54^†^	Create opportunities for students to experience research as participants	3.55	2.89
9	To give more incentives to get involved in research, for example, financial, discounts on food/drinks etc.	3.21	2.37
44	Have a regular Occupational Therapy Twitter talk for Northumbria students to ask questions and put ideas forward	3.09	2.35
17	Taking part in surveys	3.05	3.24
20	Have research competitions	2.27	2.00

Statement numbers in left hand column correspond to the numbers depicted in the concept map (Figure 1) and go-zone plots (Figure 3). Mean scores for each cluster are in bold.

† High importance/high success (upper right quadrant of the go-zone).

‡ High importance/low success (lower right quadrant of the go-zone and identified as an improvement target).

The themed clusters and their contents are reported in the order of their priority rating, with the cluster with the highest importance rating discussed first.

The ***Wider Research Community*** cluster contained 14 statements with a mean importance rating of 3.91 and a success rating of 2.98 out of 5. Within this cluster, four statements fell within the top right quadrant of the go-zone (Figure 2 and Table 1), indicating that they were both important needs and successfully met at the time that they were rated. Examples of statements falling within this quadrant were: *#3 Have patient/service user involvement in the teaching of research and #1 Encourage occupational therapy staff and students to make links with other disciplines.* Within the lower right quadrant of the go-zone, three statements were targets for improvement as they are rated as being important but having a score below the mean for current success (Figure 2 and Table 1). Statements within this quadrant were: *#69 Make research more fun; #23 Input and inspiration from local clinicians and service users to help develop research ideas* and *#15 To have talks from external OT graduates.*

The ***Integration of Research into the Core Programme*** cluster contained 28 statements with a mean importance rating of 3.82 and a success rating of 2.97. Nine statements within this cluster fell within the high importance, high success quadrant of the go-zone (Figure 2 and Table 1), which was mainly related to teaching delivery of research within the programmes. Examples include: *#31 Teaching on academic writing#47 Early use of critical appraisal tools within the programme; #21 Teach information retrieval strategies; #60 Demystify research and make it more accessible; #57 Incorporate research examples throughout all modules of the programme* and *#55 Practical tasks to learn and understand the research process.* There were also three specific targets for improvement within this cluster (Figure 2 and Table 1): *#18 Provide access to examples of previous dissertations; #13 To provide students a list of research project options to research for a dissertation* and *#50 Teach practical skills to help students learn how to design and carry out a research project.*

The ***Advanced Research Support & Capacity Building*** cluster contained 18 statements with a mean importance rating of 3.77 and a success rating of 2.64. Nine statements fell within the high-importance, high-success quadrant of the go-zone (Figure 2 and Table 1). These included *#36 Offer research drop-ins for staff and students to discuss their research ideas; #10 Research mentors for staff and students; #25 Clear signposting to research opportunities both within Northumbria University and outside of the organisation* and *#34 Hold an annual event for all students, staff and clinicians presenting current and completed projects and generating ideas for new projects.* The three targets for improvement within this cluster (Figure 2 and Table 1) included: *#35 Opportunities for practice-based research projects; #52 Inform students how they may progress to completing a PhD* and *#62 Additional support for staff and students who wish to apply for funding and awards.*

The **Research Awareness** cluster included 12 statements with a mean importance rating of 3.51 and a success rating of 2.72. There were five important and successful statements within this cluster (Figure 2 and Table 1) including: *#14 Ensure published Northumbria University research is readily accessible; #70 Organise Q&A sessions from senior researchers for staff and students;* and *#54 Create opportunities for students to experience research as participants* (Figure 2 and Table 1). The three targets for improvement in this cluster were: *#61 Discuss research careers for occupational therapists as being a viable option from year 1 of the programme; #63 OT debates to create discourse around current OT issues/research* and *#65 An in house ‘research digest’ that compiles relevant and recent research undertaken by staff/students from the occupational therapy programme.*

## Discussion and implications

We used GCM methodology to create a concept map to inform a strategy for further embedding research in the pre-registration occupational therapy programmes and enhancing the research culture at a UK HEI. The concept map developed with input from students and staff identifies how research is already successfully embedded within the programmes and identifies areas to improve this and enhance the research culture. The concept map contains four themed clusters.

The cluster named *Research Awareness* is the cornerstone of our research strategy; there is wider agreement that students should be introduced to evidence-based practice at an early stage (Bozzolan et al., 2014), and that a social constructivist approach, whereby learners construct understanding through interaction with the activities, with peers and with the wider learning environment, is as important as direct instruction (Thomas et al., 2011). A social constructivist approach also aligns with the cluster of the *Wider Research Community*, emphasising the importance of connections and collaboration. Two of the clusters – *Integration of Research into the Core Programme* and *Advanced Research Support and Capacity Building* – resonated with the idea of differentiating between universal requirements defined by baseline skills required by all to translate evidence into practice (Health Education England, 2022), and the advanced skills required when engaging in empirical research (Hitch and Nicola-Richmond, 2017).

Within the clusters, 22 ideas were rated as both high in importance and current success. The study also identified 12 specific targets for improvement that were evenly split between each of the themed clusters and can be seen in the working model of the concept map (Figure 3). This model provides the starting point of a strategy to better embed research within the occupational therapy programmes and enhance the research culture. The high-importance/low-success items in the go-zone (Figures 2 and 3 and Table 2) provide the first steps. The following steps of the strategy will consider the ideas which were deemed to be of high importance/high success (Table 2), as, overall, the success scores were consistently lower than the importance scores, demonstrating that there is still room for improvement.

Beyond interpreting the participant statements concerning areas of current success and areas of improvement, Fung’s Connected Curriculum Framework (2017) provides an interpretive lens to consider the findings in full and make connections between the clusters. Fung’s framework includes six associated dimensions of practice that emphasise the need for connectivity in individual areas. These are: ‘students connect with researchers and the institution’s research’; ‘a throughline of research activity is built into each programme’; ‘students make connections across subjects and out to the world’; ‘students connect academic learning with workplace learning’; ‘students learn to produce outputs – assessments directed at an audience’; ‘students connect with each other, across phases and with alumni’ (Fung, 2017: 5). We propose that all these dimensions can be seen in our findings and, therefore, lend support to Fung’s central tenet that the predominant mode of learning in higher education should be through active enquiry and, where possible, engagement in research.

Connections between researchers and the institution’s research are reflected in statements that propose opportunities for communication and networking (such as annual events or having access to a research digest) and statements that support the co-generation of new project ideas between staff, students and clinicians. The statement about the early use of critical appraisal tools aligns with the notion of a throughline of activity, while other statements (e.g. research supervisors for every student contrasted with additional support for those wanting to apply for funding and awards) highlight that this throughline should balance universal and targeted elements. Frequent references to desired links with clinicians, service users and other professionals, and representing different specialisms and backgrounds, are suggestive of the third dimension of making connections with other disciplines. The third dimension also emphasises outward facing connections into the world and to complex societal challenges, reflected in statements about connecting to professional priorities and contemporary professional debate.

Links to the fourth dimension, – ‘students connect academic learning with workplace learning’, can be seen in statements referring to generating topics on placement, experiencing practice-based research projects or engaging in a research internship. The fifth and sixth dimensions are further evidenced in statements concerning assignments and journal clubs linked to producing outputs and through reference to peer-learning, mentors and graduates.

However, Fung’s framework also highlights areas that were less obvious in our findings. For example, Fung presents a more detailed discussion of academic outputs that reflect a range of communication approaches for a range of audiences, such as conference posters, blogs and podcasts. Such examples of contemporary outputs are not reflected in our findings. Another gap is potentially connecting with peers at different levels of learning and across disciplines, proposed as a central part of the sixth dimension promoting student connections across phases. The importance of such ideas and their presence within the current programmes are, therefore, questioned and emphasise the significance of localised debate.

Our study has provided a local implementation plan, which has identified some specific targets to embed research in the occupational therapy curriculum and enhance the culture of research within our occupational therapy programmes. An overarching theme across participant statements and a central theme of our implementation plan is the idea of authenticity. Dewey’s notion that *‘learning takes place when students are given something to do rather than something to learn’* (Dewey, 1916: 191) chimes with our findings, with opportunities to take part in surveys, to be involved in planning and design of research and opportunities for practice-based research projects all emphasised. Furthermore, authenticity as situating learning in the context of future use (Herrington et al., 2014) can be seen in the ideas about linking professional body priorities and generating ideas for research with clinicians and service users. The importance of situated and authentic learning is also emphasised in policy drivers focused on developing research capability and capacity across the health and care workforce (Health Education England, 2022; NHS England, 2024), which stress the value of direct experience in planning, designing and delivering research to enhance outcomes for service users, emphasising its importance across all levels of practice.

This shared vision and appetite for students and staff to be connected in authentic ways to each other and to actively conduct research within the institution has already led to change and success. Staff, students and service-user contributors have increased the number of co-authored publications, for example, (Young and Collins, 2022), including those specifically raising the profile of patient and public involvement in research, for example, (Williamson et al., 2023).

While the act of asking research questions and collating project data have, in themselves, naturally contributed to changes in research conversations and culture, other developments that have happened alongside this project are also likely to have influenced cultural change. These include appointments to research leadership roles, increased numbers of research active staff and changes to student assessments with closer alignment with publication guidelines and criteria. We are aligning intentional objectives from the concept map with the more organic developments that have occurred concurrently with this research process. Making research more fun and integrating opportunities for research careers and pathways are examples of this intentional activity that will require specific objectives and measures of success. Universities have multiple aims, including providing high-quality teaching, engaging in world-leading research, meeting professional and workforce needs of contemporary society, while ensuring student experience and well-being remains central (Office for Students, 2021, 2022; UKRI, 2022). Many authors suggest that the functions of research and teaching (or teaching and research depending on emphasis) are closely linked, with the quality of Higher Education teaching being a consequence of original contributions to research (Matthews and Kotzee, 2022). However, the interconnection and integration of research and teaching are complex. Critics highlight the irony of advocating for a positive relationship between research and teaching while noting the prevalence of poor-quality research and the absence of clear evidence supporting this correlation (Tight, 2016). Tight (2016) further suggests that the bond between teaching and research needs to be nurtured locally. Our study gives a practical and co-produced example of such local nurturing. Students and academics who simultaneously engage in teaching, learning and research have contributed to a concept map forming the basis of a research strategy in which the two functions aim not only to co-exist and connect, but also to optimise outcomes, experience and opportunity for all.

Our study has some limitations. We collected limited demographic data from participants. Collating further demographic variables from participants (e.g. age and gender, home/international student status) may have enhanced the study by allowing for a more comprehensive understanding of the participant characteristics. More staff completed the sorting task than students. However, student representation from both the MSc and BSc (Hons) programmes was present, and the quantitative analyses reduced the risk of bias inherent in a purely qualitative approach. Every participant completing the sorting exercise contributed to the creation of the themed clusters, ensuring that their opinions were integrated into the co-authored concept map.

Furthermore, the stress value from the MDS was well within an acceptable range for a GCM project (Rosas and Kane, 2012), indicating the goodness of fit between the point map representations and the sorted relationships within the similarity matrix. Due to the timing of our data collection, we did not collate responses from students enrolled on the Degree Apprenticeship programme. However, our findings have provided valuable insights into this program’s delivery. Finally, student stakeholders were not involved in the statement reduction process (Stage 2). We believe that involving students at this stage may have improved the rigour in decision making around redundant statements.

The findings of this research study align with the global evidence on this topic (Helgøy et al., 2022; Helgoy et al., 2020; Riiser et al., 2023). However, we recommend further exploration related to staff and student perceptions of how well research is embedded into occupational therapy programmes and to evaluate the research culture, both in the UK and internationally. Future research may entail conducting qualitative focus groups and interviews to elicit more detailed responses after implementing any changes following this exploration. Further quantitative evaluations may include an audit using the Research Capacity and Culture Tool (Holden et al., 2012) or similar measures. Other universities and services may want to adopt a similar mixed-methods approach as we have done for their own evaluations. However, the concept map developed in this study could be utilised as a starting point for other organisations.

## Conclusion

By engaging students and staff in the occupational therapy programmes at a UK HEI using a mixed-methods approach, we have developed a co-authored concept map that informs a strategy to better embed research within the programmes and enhance the research culture among students and staff. Our strategy is based on the four identified themed clusters and starts with the specific identified improvement targets within each. The concept map and priority ideas within its themed clusters can be used as a starting point for other organisations looking to further embed research into their programmes and enhance their research culture. Finally, our approach and our subsequent findings may additionally provide valuable insights to HEIs outside of the UK.

**Key findings**

- Occupational therapy students and staff at a UK HEI aspire to join a wider research community.
- Facilitating research opportunities for students and staff through connections, engagement and collaborations are key to their development as evidence-based practitioners.

**What the study has added**

Through engaging students and staff in a combination of group processes, this study has resulted in a concept map, which informs a strategy to better embed research, set priorities and develop research culture in occupational therapy programmes at a UK HEI.
